# Supplementary material for: Synergistic elastase and papain injury drives abdominal aortic aneurysm formation and rupture in mice
Source: Commun Med (Lond). 2026 Mar 3;6:217. doi: 10.1038/s43856-026-01485-x (PMC13076655; doi:10.1038/s43856-026-01485-x)
Supplement: Supplementary file 2 — Supplementary Information [file 43856_2026_1485_MOESM2_ESM.pdf]

Supplementary Information

Synergistic Elastase and Papain Injury Drives Abdominal Aortic Aneurysm Formation and Rupture in Mice

Santiago Elizondo-Benedetto[1], Mohamed S Zaghloul[1], Batool Arif[1], Ibrahim Kuziez[1], Ryan Wahidi[1,6], Mohamed A Zayed[1-7]

[1] Division of Vascular Surgery, Department of Surgery, Washington University School of Medicine, St. Louis, MO, USA.

[2] Department of Radiology, Washington University School of Medicine, St. Louis, MO, USA.

[3] Division of Molecular Cell Biology, Washington University School of Medicine, St. Louis, MO, USA.

[4] Division of Surgical Sciences, Department of Surgery, Washington University School of Medicine, St. Louis, MO, USA.

[5] Department of Biomedical Engineering, McKelvey School of Engineering, Washington University School of Medicine, St. Louis, MO, USA.

[6] Cardiovascular Research Innovation in Surgery and Engineering Center, Department of Surgery, Washington University in St. Louis, St. Louis, Missouri

[7] Veterans Affairs St. Louis Health Care System, St. Louis, MO, USA.

Corresponding Author:
Mohamed A. Zayed, MD, PhD, MBA
660 S. Euclid Avenue
Campus Box 8109 - Surgery
St. Louis, MO, United States
E-mail: zayedm@wustl.edu

Supplementary Information

1. Supplementary Figure 1
2. Supplementary Figure 2
3. Supplementary Figure 3
4. Supplementary Figure 4
5. Supplementary Figure 5
6. Supplementary Figure 6
7. Supplementary Table 1
8. Supplementary Table 2

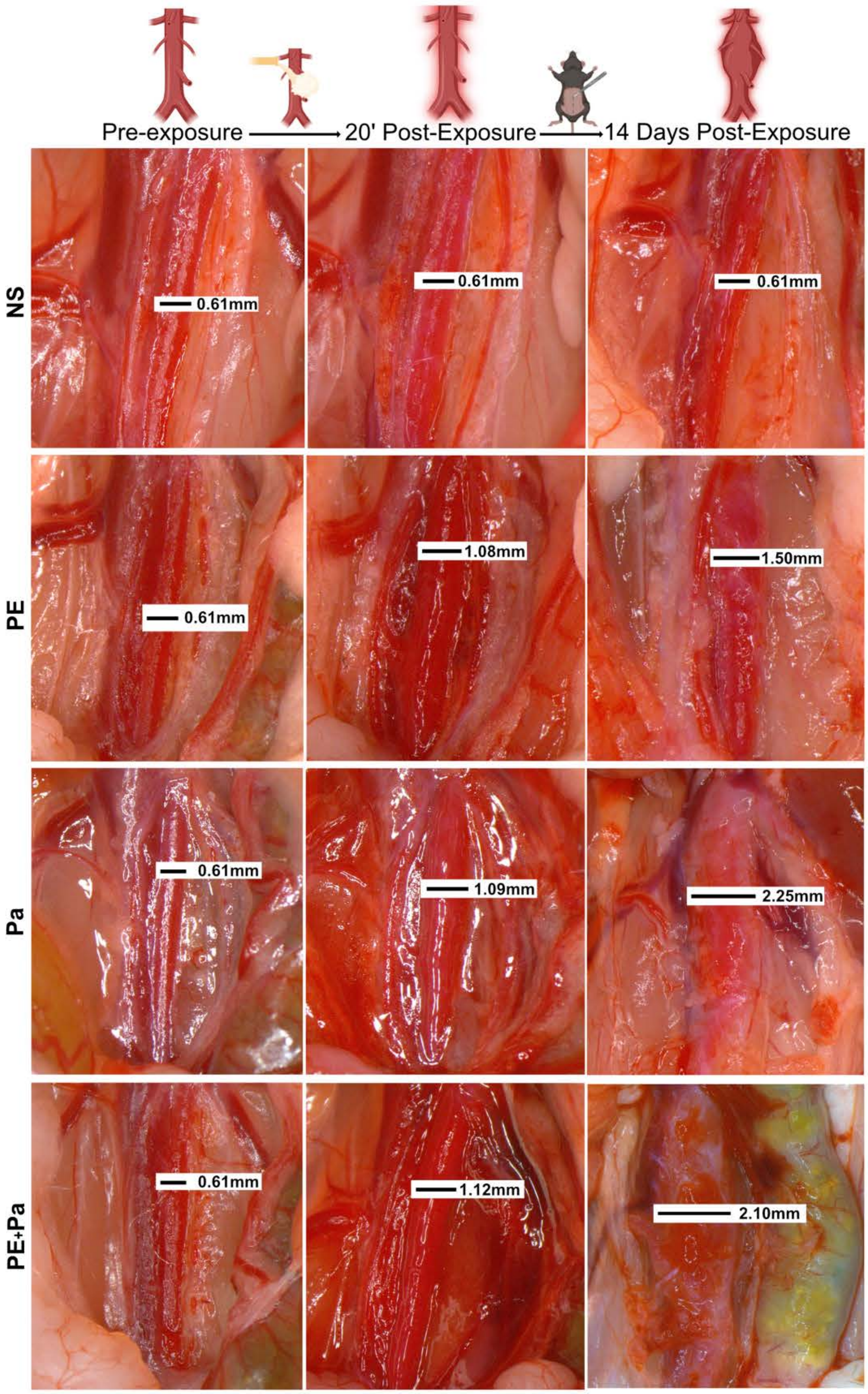

Supplementary Figure 1. Variable AAA development 20 minutes post-chemical exposure and at day 14 post-AAA formation. NS, Normal Saline. PE, Pancreatic Elastase. Pa, Papain. PE+Pa, combination of pancreatic elastase and papain.

A

	Inflammation	Elastin degradation	VSMC loss
**Mild**	No or minimal cell infiltration	Mostly intact, with less than 10% disruption	Decrease nucleus density less than 10%
**Moderate**	Scatter cells without clustering	Shredded elastin layers, 10-70% disruption	10-50%
**Severe**	Clusters of inflammatory cells	More than 70% disruption	More than 50%

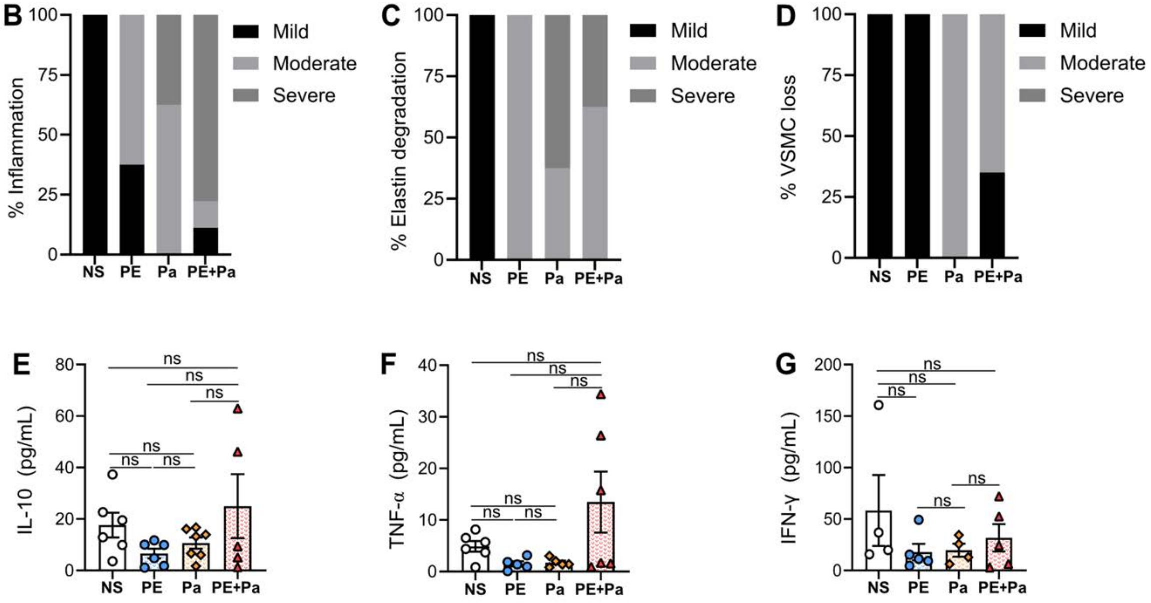

Supplementary Figure 2. Immunohistochemical categorical analysis. (**A**) Table with grading information. Three (n=3) mice of each group were harvested and processed for histopathological analysis. (**B**) Degree of inflammation, (**C**) elastin degradation and (**D**) percent of VSMC loss. A total of six (n=6) mice from the NS, PE, and PE+Pa groups, and seven (n=7) mice from the Pa group are processed for protein analysis. 1-2 tissues from NS, PE, Pa and PE+Pa were excluded due to insufficient or undetectable readings. Inflammatory markers (**E**) IL-10, (**F**) TNF-α and (**G**) IFN-γ content within the AAA tissue measured by ELISA. Data are presented as mean ± standard deviation (SD). Ns>0.05, *p<0.05, **p<0.01, ***p<0.001 using one-way ANOVA.

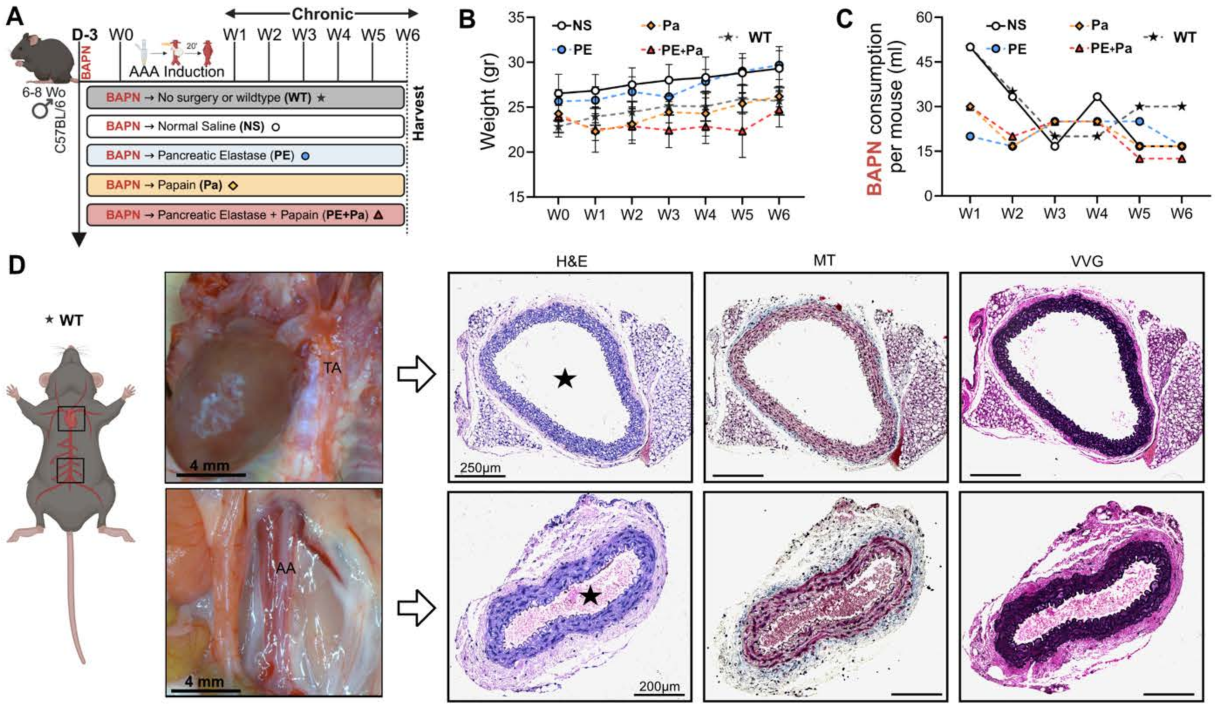

Supplementary Figure 3. Chronic model of AAA progression using daily BAPN administration over 6 weeks in wildtype (WT) mice. (**A**) WT mice (n=3) received BAPN through drinking water starting 3 days prior to AAA induction and continuing until week 6. Mice were also exposed to either normal saline (NS, n=5), pancreatic elastase (PE, n=5), papain (Pa, n=8) or a combination (PE+Pa, n=6) to promote AAA development. Panel A was made using BioRender.com. (**B**) Body weight assessment over the 6-week period, measured in grams. (**C**) BAPN consumption assessment from weeks 1 to 6. (**D**) WT mouse harvest of the thoracic aorta (TA) and abdominal aorta (AA) and stained with H&E, Masson trichrome (MT) and VVG staining (cross-section of tissue slides) with 5x magnification. The star represents the lumen of the artery. Data are presented as mean ± standard deviation (SD). Ns>0.05, *p<0.05, **p<0.01, ***p<0.001 using two-way ANOVA with multiple comparison.

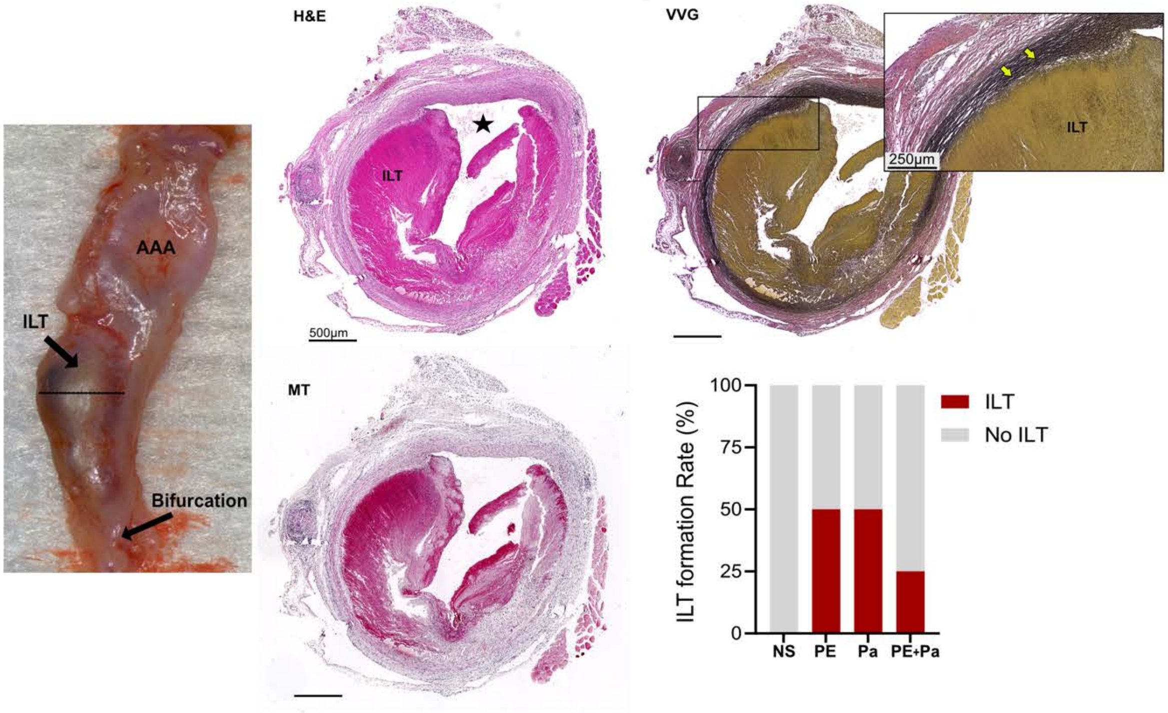

Supplementary Figure 4. Intraluminal thrombus (ILT) formation in AAA chronic model using daily BAPN administration over 6 weeks in chemically induced exposure. Surviving mice underwent histopathological ILT formation assessment: five in the NS group (0/5), four in the PE group (2/4), seven in the Pa group (with one tissue not effectively processed, 3/6), and five in the PE+Pa group (2/5). H&E, Masson trichrome (MT) and VVG staining of abdominal aortas (cross-section of tissue slides) with 5x and 10x magnification. Star = lumen. This representative picture was taken from the Pa group.

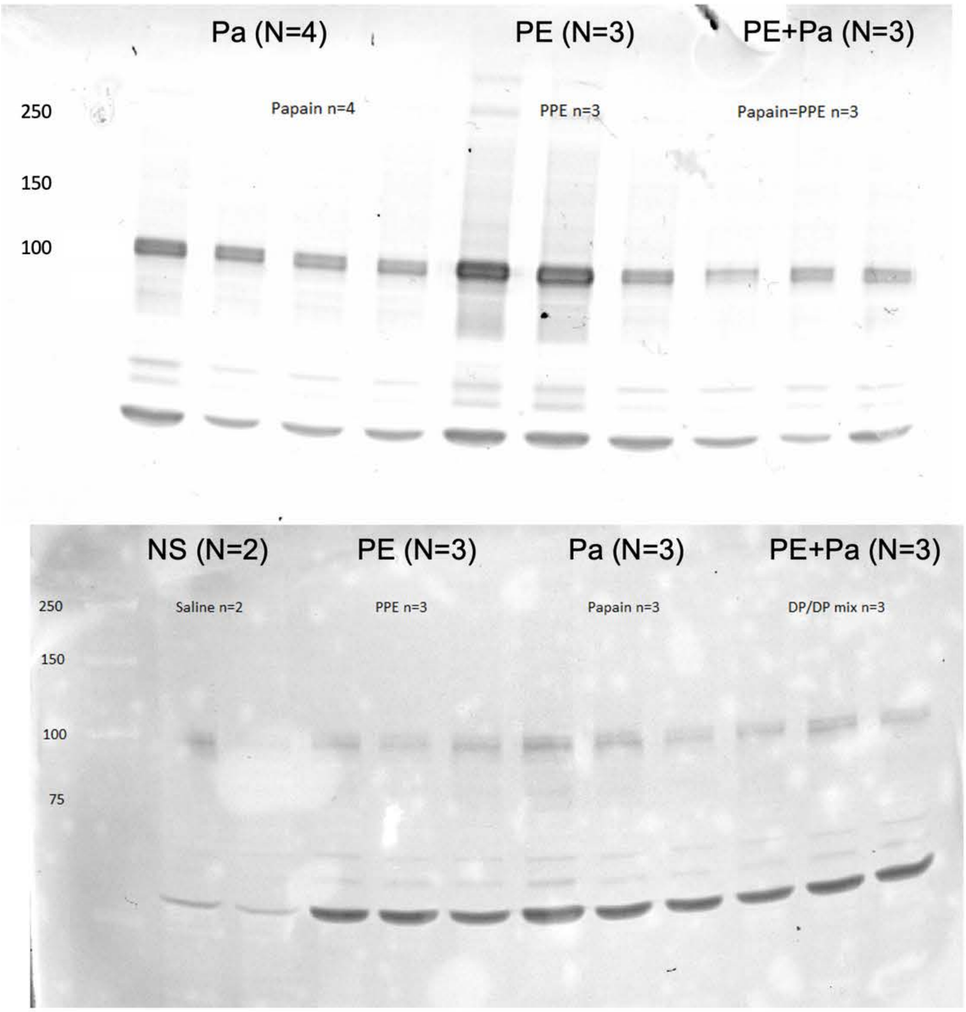

Supplementary Figure 5. Gel Zymography for Day 14 AAA formation following peri-adventitial aortic exposure to either PE, Pa or the PE+Pa combination. This raw zymography gel corresponds to Figure 1H in the main manuscript, specifically assessing MMP9 and MMP2 enzyme activity. Normal Saline (NS) was used for comparison. Analysis of each specific band was performed using ImageJ. The quantification for NS in the main figure is the result of summing the NS band lengths from this gel and Supplementary Figure 6. NS = Normal Saline, PE = Pancreatic Elastase, and Pa = Papain. Labels on the left indicate the protein sizes in kDa (75, 100, 150, and 250).

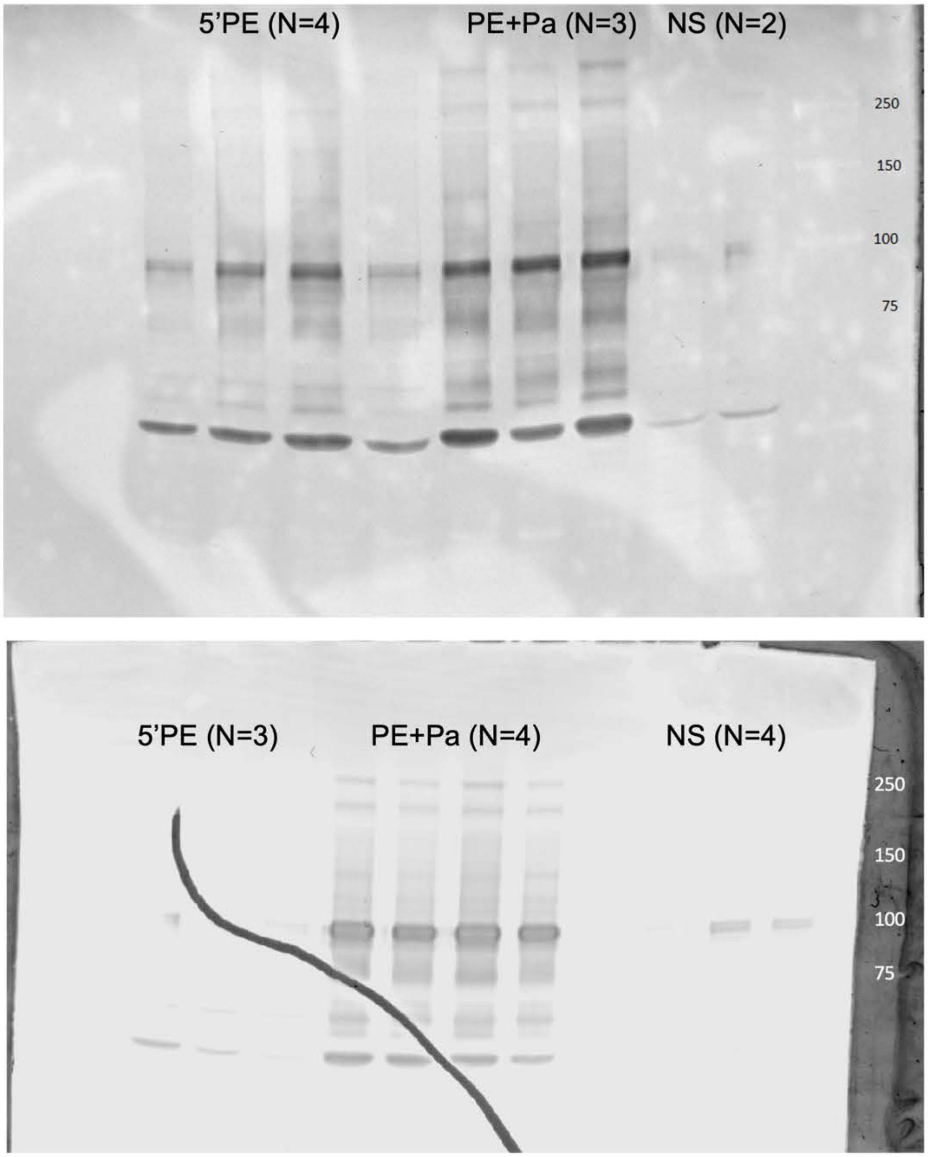

Supplementary Figure 6. Gel Zymography for Day 6 AAA formation following peri-adventitial aortic exposure to either 5 minutes of PE (5’PE) or the PE+Pa combination. This raw zymography gel corresponds to Figure 4E in the main manuscript, specifically assessing MMP9 and MMP2 enzyme activity. Normal Saline (NS) was used for comparison. Analysis of each specific band was performed using ImageJ. Labels on the left indicate the protein sizes in kDa (75, 100, 150, and 250).

Supplementary Table 1. Surgical Checklist divided by category

Category	*Equipment*	*Uses*
PPE	Surgical drapes, gauzes, gloves, gown and googles	Sterile technique and personal protection equipment
Setup	Small animal surgery board:<br>→Magnetic towers<br>→Self-retractors<br>→Hose holder<br>→3M tape	Standardized surgical technique and a comfortable setting for AAA creation surgery to ensure reproducibility
	Heating pad	Thermoregulation and maintaining adequate body temperature to enhance recovery
Anesthesia	Nose cone, parafilm and hoods<br>Oxygen 100%	Minimizes the risk of anesthesia leakage and accidental user inhalation, ensures that the anesthesia is effectively delivered to the mouse
	Isoflurane	Halogenated, volatile anesthetic
Aseptic Skin Preparation	Nair	Hair removal for the surgical preparation
	Antiseptic swabs<br>→Chlorhexidine gluconate<br>→Isopropyl alcohol	Combination of skin antisepsis used in preoperative skin preparations and surgical procedures
AAA Creation	Pipet and cotton ball	50μL pipet to drop chemical for aortic exposure to the retroperitoneal cavity through the cotton ball
	Porcine pancreatic elastase	PPE; 10.3mg protein/mL, 5.9 U/mg protein obtained from Sigma Aldrich
	Papain	Pap; 1.0 or 20mg/mL
	BAPN preparation	β-aminopropionitrile (BAPN) administration through drinking water (0.3% BAPN in water)
	Angiotensin II subcutaneous pump	Pump; Alzet 1004, Durect Corp, Cupertino, CA<br>ANG II; Sigma Aldrich Inc, St. Louis, MO

Syringes	30 G (1 ½) 0.3mm x 13mm<br>(Insulin Syringe)	Blood draw<br>Buprenorphine administration
Analgesics	Buprenorphine SR	Pre-surgical procedure analgesia
Silks	5.0 Vicryl	Uninterrupted suture closure of the rectus abdominis muscle (anterior abdominal wall)
	5.0 Monofilament Nylon	Interrupted suturing technique for skin wound closure
Hemostasis	Small Q-tips<br>Small Gauzes (2" x 2" → 5.1x5.1cm)	Rapid control of bleeding by applying pressure
Surgical Instruments	Atraumatic tissue forceps	Skin manipulation
	Fine forceps	Retroperitoneal access and aortic and vena cava manipulation
	Mayo scissors	Laparotomy
	Fine scissors	Muscle incision, silk cut and tissue management
	Needle holder	Skin and muscle suturing and closure

Supplementary Table 2. Group models of AAA Development and Rupture (Extended Information)

	Group: Topical Exposure (µL) Additional Exposure	Days	Ao Diameter (mm)		ECM Degradation		VSMC Loss	Inflammation	ILT (%)	Cytokines	Chemokines	MMP9	MMP2	Rupture Rate (%)
			Post-exposure	Harvest	Elastin	Collagen				IL-6, IL-1β, IL17A	MCP-1, RANTES			
**AAA**	**NS: Saline (50)**	14	0.5 ± 0.05	0.5 ± 0.07	N	N	Mild	Mild	0%	N	N	N	N	0%
	**PE: Pancreatic Elastase (50)**	14	0.9 ± 0.13	1.4 ± 0.3	↑↑	↑	Mild	Moderate	0%	↑	↑	↑	N	0%
	**Pa: Papain (50)**	14	1.0 ± 0.2	1.8 ± 0.2	↑↑↑	↑	Moderate	Moderate	0%	N	↑↑	↑↑	N	0%
	**PE+Pa: PE (50) / Pa (50)**	14	1.0 ± 0.07	1.7 ± 0.3	↑↑↑	↑	Moderate	Severe	0%	↑↑	↑↑↑	↑	N	0%
**Chronic AAA**	**WT: No exposure BAPN**	42	N/A	0.53 ± 0.1	N	N	Mild	Mild	0%	N/A	N/A	N/A	N/A	0%
	**NS: Saline (50) BAPN**	42	0.5 ± 0	0.56 ± 0.0	N	N	Mild	Mild	0%	N/A	N/A	N/A	N/A	0%
	**PE: Pancreatic Elastase (50) BAPN**	42	0.8 ± 0.05	4.7 ± 1.2	↑↑↑	↑↑	Severe	Moderate	50%	N/A	N/A	N/A	N/A	20%
	**Pa: Papain (50) BAPN**	42	1.15 ± 0.3	4.3 ± 1.3	↑↑↑	↑↑↑	Severe	Moderate	50%	N/A	N/A	N/A	N/A	0%
	**PE+Pa: PE (50) / Pa (50) BAPN**	42	1.3 ± 0.08	5.1 ± 0.7	↑↑↑	↑↑↑	Severe	Moderate	25%	N/A	N/A	N/A	N/A	17%
**Rupture AAA**	**5'PE: Pancreatic Elastase (5) * BAPN**	14	0.5 ± 0.05	1.4 ± 0.4	N/A	N/A	N/A	N/A	N/A	N/A	N/A	N/A	N/A	0%
		6	0.5 ± 0.04	1.0 ± 0.4	↑↑				0%	↑	↑	N	N	
	**PE: Pancreatic Elastase (50) BAPN + ANG II**	14	0.8 ± 0.07	N/A	N/A	N/A	N/A	N/A	N/A	N/A	N/A	N/A	N/A	60%
	**Pa: Papain (50) BAPN + ANG II**	14	0.9 ± 0.06	N/A	N/A	N/A	N/A	N/A	N/A	N/A	N/A	N/A	N/A	20%
	**PE+Pa: PE (50) / Pa (50) BAPN + ANG II**	14	0.9 ± 0.1	N/A	N/A	N/A	N/A	N/A	N/A	N/A	N/A	N/A	N/A	93%
		6	0.9 ± 0.1	1.3± 0.2	↑↑				0%	↑↑	↑↑↑	↑↑↑	↑↑	

*5'PE: is the only model with 5 minutes topical exposure without a cotton ball.
N/A Non-Applicable or Not Available
